# To the Editors of the Medical and Physical Journal

**Published:** 1807-04

**Authors:** M. Hall

**Affiliations:** Oxford


### *To the Editors of the Medical and Physical Journal.*

GENTLEMEN,

THE judicious remarks of Dr. Fothergill, in the last Number of your very instructive and useful Journal, on the treatment of the wound inflicted by the bite of a mad dog, induce me to request you, to recall to the attention of your readers the late excellent Dr. Percival's proposed method of washing the wound immediately and perseveringly with water. It is probable, whatever may be the nature or chemical qualities of the virus in this instance, that it is soluble in, or minutely miscible with, water. At least we certainly know, that saliva, which is the vehicle of it, is so. It should therefore be publickly made known and recommended, that persons who may suffer this dreadful misfortune, should immediately apply to the first spring, brook, pool, or ditch, which can be found, and of all remedies there is fortunately none so immediately within reach and command. Even if the injury be received at a distance from any town or village, water is always near, and the part may be easily cleansed. If the accident happen in the neighbourhood of any house, the water may be introduced with more force by pouring it from a cup with a spout, a tea kettle, or more forcibly still by a pump: And a sponge, &c. may possibly be obtained. A syringe, if at hand, may also be found a very useful instrument. Cold water may be used, until warm can be produced. The wound, if any friend or attendant be at hand, should be held open and dilated, that the affused or injected fluid may penetrate every part and perfectly cleanse the whole. This method does not preclude the use of chirurgical means. If a surgeon be on the spot, excision should be practiced, or the actual cautery applied directly. I have seen it asserted in some publication, that upon occasion of such an accident, in any situation, every one who carries a penknife, or even a common pocket knife, may be his own surgeon. But such an operation is not easy for one unpracticed in surgery, even if he have sufficient firmness of mind to attempt it; the wound is often inflicted in parts, such as the hand, where there is but little muscular flesh, and only a congeries of tendons, nerves, and blood vessels. An ignorant person might by a knife, unskilfully used, bring on very alarming symptoms. Therefore during ring that interval of time, while a surgeon is sought for, the method of ablution here recommended may be diligently and assiduously practiced. As in the use of the means for the recovery of suspended animation, so in this instance, are required unremitting energy and perseverance.

I believe this practice was first suggested by Dr. Percival, at the end of the annual statement of the Manchester Infirmary, about twelve or fourteen years ago. The late Earl of Macclesfield desired it might be annexed to the statement of the Radcliffe Infirmary; and Dr. Percival having written to me on the subject, I printed several hundred copies of the letter, to be distributed gratis about the country, and stuck up in market-places, public houses, and at the church doors in the villages of this neighbourhood. I have since communicated it by correspondence to some parts of Scotland, and am very anxious it should be generally known. I am, &c.

M. HALL.

*Oxford, February 26,* 1807.

---

